# Diagnostic Accuracy of the Abbott SD Bioline MPT64 antigen test for identification of MTB Complex in a U.S. Clinical Mycobacteriology Laboratory

**DOI:** 10.1016/j.heliyon.2024.e30501

**Published:** 2024-04-30

**Authors:** D.T. Armstrong, Logan Pretty, Karen D'Agostino, Roxanne Redhead-Harper, Nicole Parrish

**Affiliations:** Johns Hopkins Hospital, School of Medicine, Baltimore, MD, USA

**Keywords:** MPT64, Antigen, Culture, Mycobacteriology, Tuberculosis

## Abstract

Identification of the *Mycobacterium tuberculosis* complex (MTBC) from culture and differentiation from other non-tuberculous mycobacterial species is required for rapid diagnosis and accurate treatment. However, gaps exist in culture-based identification of acid-fast bacilli (AFB) positive cultures for rapid rule-out of MTBC in the United States. The SD Bioline™ MPT64 (Abbott Inc, South Korea) lateral flow assay (LFA) has high sensitivity and specificity for the detection of MTBC in liquid culture but has not been evaluated in a clinical mycobacteriology laboratory in the United States. We conducted a diagnostic accuracy study of this LFA for detection of MTBC versus NTMs on AFB positive cultures. A total of 362 tests were performed, with a sensitivity and specificity of 100 % (362/362) across all tests. The SD Bioline MPT64 assay provides accurate test results with AFB-positive liquid cultures and could fill the current gap for rapid rule-out of MTBC in U.S.-based clinical laboratories.

## 1 Introduction

According to a 2022 World Health Organization (WHO) report tuberculosis (TB) is the second leading cause of death from infectious disease, with an estimated 10.6 million infections and claiming the lives of around 1.6 million people annually [1]. Approximately 87 % of new TB cases come from high burden countries, with rates in the U.S. reported at around 8000 cases in 2022 [2,3]. Rapid detection of the *Mycobacterium tuberculosis* complex (MTBC) allows for not only prompt placement of patients in isolation with concurrent contact tracing but also initiation of appropriate treatment. For these reasons, accurate and rapid laboratory diagnostics are imperative to lower the TB burden around the globe. In the United States the detection of MTBC from acid-fast bacilli (AFB) positive cultures has relied to a large extent on the recently discontinued Accuprobe™ platform (Hologic Inc., San Diego, CA), which provided for same-day identification of not only MTBC but also three species of non-tuberculous (NTM) mycobacteria (M. *avium complex*, M. *kansasii* and M. *gordonae*). For the past couple of decades, the Accuprobe assay has been the only FDA-approved platform for culture-based identification of the MTBC, and has demonstrated excellent sensitivity and specificity [3,4]. Since the discontinuation of this assay in December 2022, U.S. based clinical mycobacteriology laboratories have had to identify and validate other platforms to fill this gap [5]. Other non-FDA approved methods for rapid identification directly from positive cultures include the GeneXpert™ MTB/RIF assay (Cepheid, Sunnyvale, CA) whole-genome sequencing and other molecular lab-developed tests, many of which can be expensive, requiring additional resources and leading to an increase in turnaround times for reporting. A rapid, low-cost diagnostic test would be ideal to fill this current gap in the United States-based TB diagnostic algorithms.

The SD Bioline MPT64 Ag test (Standard Diagnostics, Abbott Inc, South Korea)is a rapid lateral flow assay that uses mouse monoclonal anti-MPT 64 antibodies to detect the presence of MTBC in liquid culture media such as the MGIT- 960 within 15 min [6]. The platform has been rigorously evaluated in international settings, with an overall sensitivity and specificity >99 % [[7], [8], [9]]. However, this test has not been evaluated in a clinical setting in the United States and given the current gaps in culture-based identification could prove to be a useful tool. Therefore, the purpose of this study was to determine the sensitivity and specificity of the SD Bioline MPT64 antigen test for identification of MTBC directly from MGIT-960 broth cultures in a large clinical mycobacteriology laboratory.

## 2 Materials and methods

This study took place in the Johns Hopkins Clinical Mycobacteriology Laboratory from 2021 to 2022, and was approved by the Johns Hopkins University Institutional Review Board (IRB). For all assays, SD Bioline MPT64 kits were donated by Abbott and testing was performed solely for research use only according to the Package Insert.

### 2.1 Testing consisted of two phases

Phase I was a retrospective evaluation using MGIT-960 tubes spiked with both clinical and reference isolates of MTBC and NTM with subsequent determination of sensitivity and specificity. Multiple lots of MGIT-960 tubes were used and the limit of detection (LOD) determined.

The MTBC and NTM strains used in this study came from both the Johns Hopkins clinical strain repository as well as the American Type Culture Collection (ATCC, Manassas Virginia) and included a variety of both drug-susceptible and drug-resistant MTBC cultures across multiple Lineages (1–5, data not shown). Isolates had been previously identified using routine identification methods available in the laboratory, including the Accuprobe ™ platform, matrix-assisted laser desorption/ionization-time of flight mass spectrometry (MALDI-TOF MS), 16S rRNA sequencing, GenoScreen Deeplex ™ assay, and GeneXpert™ MTB/RIF. For spiking studies, isolates were removed from the freezer, thawed and cultured onto Lowenstein-Jensen media (ThermoFisher, Lenexa, Kansas) and incubated at 30 °C–37 °C with and without CO_2_ based on individual species requirements. Subsequently, a swab full of visible growth was inoculated into a fresh MGIT-960 tube to create a heavy inoculum (confirmed as greater than a McFarland 0.5 standard), vortexed, and then 100 μL placed onto the SD Bioline lateral flow device. To simulate a mixed-culture, a subset of MGIT-960 tubes were also spiked with either the MTBC and NTM's along with other bacteria (either *Staphylococcus aureus* or *Escherichia coli*) at a 0.5 McFarland and subsequently tested using the SD Bioline.

To assess detection on the MGIT-960 platform, a set of 10 cultured samples (including both MTBC and NTM) previously grown on Lowenstein-Jensen media were spiked into MGIT tubes using standard procedures for liquid culture inoculation and tested on the MGIT system. When tubes flagged positive for growth, AFB smears were made (to confirm positivity) and the SD Bioline MPT 64 test was performed, according to manufacturer's instructions. The limit of detection was determined using a suspension from a clinical TB isolate (CAM144) prepared in Middlebrook 7H9 broth (M7H9) and adjusted to a 0.5 McFarland standard. Subsequent 10-fold dilutions were prepared in sterile water, with final concentrations ranging from 10^7^ to 10^1^ cfu/mL. Testing on the SD Bioline was performed in duplicate and the results recorded. Multiple uninoculated MGIT-960 tubes (n = 20) representing 3 different lots were also tested using the SD Bioline as negative controls.

In Phase II, prospective testing occurred alongside routine culture work-up to assess the utility of the SD Bioline test for use with AFB positive, MGIT-960- cultures with assessment of incorporation into an existing laboratory testing algorithm. Briefly, once the MGIT-960 instrument flagged positive, routine identification ensued with the SD Bioline assay performed simultaneously and the results compared. All technologists performing the SD Bioline assay were blinded to test results obtained using standard clinical methods.

## 3 Results

A total of 355 MGIT-960 cultures were tested from October 2021 to May 2022, with 107 tests performed in Phase I and 248 in Phase II. All tests conducted (100 %, 355/355) provided determinate results on the assay. A break-down of results are shown in Table 1.

**Table 1:** Total number of tests performed for phase I and phase II.

Category	# of Tests	Organism (#)	MPT64 (+)	MPT64 (−)
MTBC	47	M. *tuberculosis complex* (47)	47/47 (100 %)	0/0 (100 %)
NTM	25	M. *scrofulaceum* (1) M. *kansasii* (1) M. *avium complex* (8) M. *xenopi* (1) M. *wolynski* (1) M. *malmoense* (1) M. *arupense* (1) M. *chimaera* (1) M. *terrae complex* (1) M. *gordonae* (3) M. *haemophilum* (1) M. *houstonese/senegalense* (1) M. *smegmatis* (1) M. *franklinii* (1) M. *mucogenicum* (1) M. *fortuitum* (1)	0/0 (100 %)	25/25 (100 %)
MTBC + NTM	3	M. *tuberculosis complex* + M. *fortuitum* (1) M. *tuberculosis complex* + M.*peregrinum* (1) M. *tuberculosis complex* + M. *kansasii* (1)	3/3 (100 %)	0/0 (100 %)
MTBC + Bacteria	5	M. *tuberculosis complex* + S. *aureus* (2) M. *tuberculosis complex* + E. *coli* (3)	5/5 (100 %)	0/0 (100 %)
Uninoculated MGIT	20	NA	0/0 (100 %)	20/20 (100 %)

### 3.1 Phase I

Twenty uninoculated MGIT tubes from 3 different lots were tested, with 20/20 (100 %) shown to be MPT64 negative. A total of 47 tubes were spiked with MTBC only, with 47/47 (100 %) providing MPT64 positive results. All tubes spiked with various NTM species (25/25, 100 %) were found to be MPT64 negative. For the 10 tubes spiked with either MTBC or NTM and run on the MGIT-960, all flagged positive (confirmed AFB by smear) and provided expected MPT64 results. MGIT tubes that contained both MTBC and bacteria provided 100 % positive MPT64 results (5/5), and all MGIT tubes spiked with both MTBC and NTM were positive for MPT64 (3/3, 100 %). A summary of results is shown in Table 2.

**Table 2:** Phase I clinical testing Results-MGIT (N = 100).

MTBC Bacillary Load (cfu/mL)	MPT64 Result
1x10^7	(+)
1x10^6	(+)
1x10^5	Faint band (+)
1x10^4	Very faint band (Weak +)
1x10^3	(−)
1x10^2	(−)
1x10^1	(−)

The limit of detection of the SD Bioline assay was determined using MGIT-960 tubes spiked with an MTBC strain (CAM144) at 7 different final concentrations ranging from 10^7^ cfu/mL to 10^1^ cfu/mL. Strong positive bands were confirmed in samples with mycobacterial concentrations at 10^7^ and 10^6^ cfu/mL. Weak bands that were still considered positive were observed at 10^5^ and 10^4^ cfu/mL, with the MPT64 test providing negative results at 10^3^ cfu/mL and lower concentrations. Results are shown in Table 3.

**Table 3:** Phase I limit of detection results (TB isolate CAM144).

MGIT Culture Final ID	# Tested	MPT64 (+)	MPT64 (−)
**NTM** M*. avium complex* M. *abscessus* M. *fortuitum complex* M. *kansasii* *M. chelonae* *M. peregrinum* M. *gordonae* M*. lentiflavum* M. *mageritense* M. *mucogenicum* M. *mantenii* M*. simiae*	**157** (94) (26) (15) (13) (2) (1) (1) (1) (1) (1) (1) (1)	0/0 (100 %)	157/157 (100 %)
**MTBC**	26	26/26 (100 %)	0/0 (100 %)
No Growth/No Organism Seen	65	0/0 (100 %)	65/65 (100 %)

### 3.2 Phase II

A total of 248 prospective samples flagging MGIT positive were tested on the SD Bioline MPT64 assay. Of these, 157/248 (63.3 %) were classified as NTM, with 94/248 (37.9 %) having a final identification as *Mycobacterium avium* complex. The remaining NTM's included 26/248 (10.5 %) identified as M. *abscessus*, 15 (6.0 %) identified as M. *fortuitum,* 13/248 (5.2 %) identified as M*. kansasii* and 9/157 (5.7 %) identified as other less common non-tuberculous mycobacteria*.* All MPT64 tests performed on these MGIT cultures (157/157, 100 %) provided a correct result of MPT64 negative. An additional 26/248 (10.5 %) were identified as MTBC, which included 15 individual patients confirmed with MTBC infection and 11 additional duplicate cultures tested for reproducibility. All 26/26 (100 %) provided MPT64 positive results. An additional 65 MGIT tubes (26.2 %) flagged but had a final result of negative after a full 6-week incubation (Smears showed no organism seen). All of these (65/65, 100 %) were correctly identified as MPT64 negative. A breakdown of results is shown in Table 4.

**Table 4:** Phase II prospective testing results.

Category	# of Tests
Phase I: Spiked MGIT Tubes Limit of Detection (LOD) Testing Uninoculated MGIT tubes (Multi-Lot Testing)	80 7 20
Phase II: Prospective Testing	248
Total Number of Tests	355

## 4 Discussion

Rapid diagnosis for the detection of the MTBC in AFB-positive liquid cultures is critical, even in a low-prevalence setting for tuberculosis, such as the United States. For more than 2 decades, many clinical and public health laboratories have relied on the Accuprobe™ platform for rapid identification of the MTBC directly from AFB-positive cultures. However, the discontinuation of this test from the commercial market has created a need for a rapid, low-cost diagnostic that could not only identify the MTBC, but do so the same day the culture flags positive.

The SD Bioline test can distinguish the MTBC from NTM in AFB-positive MGIT-960 broth cultures in 15 min and is routinely used outside the United States in both clinical and public health mycobacteriology laboratories [[7], [8], [9]]. Recently, the World Health Organization has reiterated the need for early and rapid diagnosis of TB, making tests like the SD Bioline a critical part of this algorithm [10].

This study sought to evaluate the SD Bioline test to determine its utility in a large, clinical mycobacteriology laboratory for rapid identification of the MTBC from positive MGIT-960 cultures. We tested 355 MGIT cultures across two phases, performing both retrospective testing on spiked MGIT cultures as well as assessing real-time utility of testing alongside routine clinical work-up in the laboratory.

Testing in Phase 1 showed 100 % positivity for all spiked MTBC strains, as well as confirmed negativity for all spiked NTM cultures. Testing included MTBC strains with varying drug-resistant profiles ranging from Pan-susceptible to extremely drug-resistant (XDR) as well as Strains from Lineage 1–5 (data not shown). In previous reports it has been shown that Lineage 5 MTBC strains from West Africa have a decreased sensitivity on MPT64 tests [11]. However, amongst our strains tested we had 1 Lineage 5 strain, which was MPT64 positive.

To assess cross-reactivity and specificity, 16 different NTM species (both slow and rapid growing) were tested across 25 samples; all results were MPT64 negative. When MTBC was mixed with NTM or bacteria (both spiked with >0.5 McFarland), results showed 100 % positivity. In addition, there were no false positives when testing uninoculated MGIT tubes. This agrees with previously published data [7,8].

In verification testing of the Limit of Detection (LOD), we observed strong positive bands down to 1x10^6^ cfu/mL, but a faint band at 1x10^5^ cfu/mL. At a concentration of 1x10^4^ cfu/mL the band was extremely faint, with the LOD likely calculated between these two concentrations. This coincides with previous data stating the LOD is approximately 6.9x10^4 cfu/mL [6]. Since the limit of detection of smear positivity is approximately 1x10^4^ cfu/mL, it is reasonable to conclude that this test provides a sensitive and accurate identification for flagged MGIT cultures that are found to be AFB smear-positive after AFB microscopy is performed. The threshold of the MGIT-960 is typically around 10^6^ cfu/mL for detection, therefore use of the SD Bioline on AFB-positive MGIT cultures is likely to provide a determinate result.

In Phase II testing, 248 MGIT cultures were tested in real-time, after routine culture identification occurred. Testing on cultures with a final identification of NTM were all found to be MPT64 negative. In our laboratory, most AFB-cultures are identified as NTM, with nearly 60 % comprised of the M. *avium complex*. With the discontinuation of the AccuProbe™, identification must now be performed using a variety of alternate methods including MALDI-TOF MS or various sequencing modalities. Ultimately, what is needed is a rapid, same day test which can rule out MTBC from AFB positive cultures. Such a test would be especially useful in patient populations with chronic NTM disease to distinguish MTBC providing for initiation of appropriate treatment and isolation for those with tuberculosis.

The SD Bioline AG MPT64 test not only provided rapid results from an AFB positive culture within 15 min but also is relatively low in cost, potentially 5–10 USD per test [11]. This is an important consideration given the financial constraints for many laboratories. In comparison, rapid identification of the MTBC from positive cultures using alternate methods can be > 50 USD for more sophisticated molecular methods. Costing studies showing the impact of this rapid diagnostic in routine testing should be performed if this test is able to be implemented in the U.S. setting. In addition, other MPT64 testing platforms should be evaluated in this setting to determine comparability of results.

This study was not without limitations. There were no MPT64 negative MTBC isolates identified in this study. As noted, there are strains of MTBC (such as Lineages 5 and 6) where expression of the *mpt64* gene is low, therefore giving a false negative for this assay [12]. These two lineages of MTBC are most prevalent in West Africa and may not be as commonly seen in the United States. Certain strains of BCG may also test negative [13]. In patients with MPT64 negative results where clinical MTBC is highly suspected, utilizing an alternative testing platform such as the GeneXpert MTB/RIF assay could provide confirmation of the diagnosis. Additionally, since our clinical laboratory does not see many MTBC cases, the results as described in this study may not reflect testing in other United States laboratories with a higher prevalence of tuberculosis. Our study evaluated the performance on commonly encountered and clinically relevant NTM's present in the laboratory, but was not exhaustive given there are over 200 species of NTM that have been identified. Further studies on additional species should also be performed to determine whether other species could give false results. However, this study does provide evidence that the SD Bioline MPT64 antigen test, could fill the current gap in rapid identification of MTBC from smear positive MGIT cultures. This test could provide relatively, low-cost, same-day testing for rapid rule out of MTBC in regions with a high NTM prevalence as an alternative to current methods of culture identification. Although not currently available in the United States, it is distributed in Canada. Perhaps expansion into the United States could be considered for the future.

## Additional Information

This research did not receive any specific grant from funding agencies in the public, commercial or not-for-profit sectors. The authors declare no competing interests.

## Data Availability Statement

Data will be made available upon request.

## Ethics declaration

This study was reviewed and approved by the Johns Hopkins Institutional Review Board, with approval IRB00284173.

## CRediT authorship contribution statement

**D.T. Armstrong:** Writing – review & editing, Conceptualization. **Logan Pretty:** Writing – original draft, Investigation. **Karen D'Agostino:** Investigation. **Roxanne Redhead-Harper:** Visualization, Investigation. **Nicole Parrish:** Writing – review & editing, Supervision.

## Declaration of competing interest

The authors declare that they have no known competing financial interests or personal relationships that could have appeared to influence the work reported in this paper.The authors declare the following financial interests/personal relationships which may be considered as potential competing interests: Derek Armstrong reports supplies were provided by Abbott, North America LLC. If there are other authors, they declare that they have no known competing financial interests or personal relationships that could have appeared to influence the work reported in this paper.
